# Medialization thyroplasty versus injection laryngoplasty: a cost minimization analysis

**DOI:** 10.1186/s40463-017-0191-5

**Published:** 2017-02-20

**Authors:** Samantha Tam, Hongmei Sun, Sisira Sarma, Jennifer Siu, Kevin Fung, Leigh Sowerby

**Affiliations:** 1 Department of Otolaryngology-Head and Neck Surgery, Schulich School of Medicine and Dentistry, Western University, London, ON Canada; 2 Ivey Business School, Western University, London, ON Canada; 3 Department of Epidemiology and Biostatistics, Schulich School of Medicine and Dentistry, Western University, London, ON Canada; 4 Department of Otolaryngology-Head and Neck Surgery, University of Toronto, Toronto, ON Canada; 5 St. Joseph’s Health Care Centre, 268 Grovesnor Street, London, ON N6A 4V2 Canada

**Keywords:** Vocal fold paralysis, Injection laryngoplasty, Medialization thyroplasty, Cost analysis

## Abstract

**Background:**

Medialization thyroplasty and injection laryngoplasty are widely accepted treatment options for unilateral vocal fold paralysis. Although both procedures result in similar clinical outcomes, little is known about the corresponding medical care costs. Medialization thyroplasty requires expensive operating room resources while injection laryngoplasty utilizes outpatient resources but may require repeated procedures. The purpose of this study, therefore, is to quantify the cost differences in adult patients with unilateral vocal fold paralysis undergoing medialization thyroplasty versus injection laryngoplasty.

**Study design:**

Cost minimization analysis conducted using a decision tree model.

**Methods:**

A decision tree model was constructed to capture clinical scenarios for medialization thyroplasty and injection laryngoplasty. Probabilities for various events were obtained from a retrospective cohort from the London Health Sciences Centre, Canada. Costs were derived from the published literature and the London Health Science Centre. All costs were reported in 2014 Canadian dollars. Time horizon was 5 years. The study was conducted from an academic hospital perspective in Canada. Various sensitivity analyses were conducted to assess differences in procedure-specific costs and probabilities of key events.

**Results:**

Sixty-three patients underwent medialization thyroplasty and 41 underwent injection laryngoplasty. Cost of medialization thyroplasty was C$2499.10 per patient whereas those treated with injection laryngoplasty cost C$943.19. Results showed that cost savings with IL were C$1555.91. Deterministic and probabilistic sensitivity analyses suggested cost savings ranged from C$596 to C$3626.

**Conclusions:**

Treatment with injection laryngoplasty results in cost savings of C$1555.91 per patient. Our extensive sensitivity analyses suggest that switching from medialization thyroplasty to injection laryngoplasty will lead to a minimum cost savings of C$596 per patient. Considering the significant cost savings and similar effectiveness, injection laryngoplasty should be strongly considered as a preferred treatment option for patients diagnosed with unilateral vocal fold paralysis.

## Background

The aim of treatment for unilateral vocal fold paralysis (UVFP) is, firstly, to decrease aspiration, and secondly, to improve voice quality. One treatment paradigm is medialization of the paralysed vocal fold to allow for contact with the mobile vocal fold. Two options for medialization include Type 1 medialization thyroplasty (MT) and injection laryngoplasty (IL). MT, as described by Isshiki et al.*,* is considered the gold standard treatment and involves permanent medialization of the vocal fold with an alloplastic stent in the paraglottic space [1]. However, with the development of reliable injectable soft tissue fillers and distal chip flexible endoscopes, office-based IL has become a new alternative [2]. An injectable filler, such as methylcellulose, collagen, or calcium hydroxylapatite, is used to medialize the vocal fold via percutaneous or transoral injection.

Since MT and IL utilize the same medialization treatment paradigm, these techniques are often applied in similar clinical scenarios. Outcomes between treatment options have been compared and have been found to yield similar clinical outcomes [3–6]. MT offers a permanent solution but requires operating room time and sedation. In contrast, IL is performed in the outpatient clinic setting. However, the soft tissue fillers are resorbed over time and treatment may require multiple injections over one’s lifetime [7].

Given the current fiscal constraints in our health care system, the cost of both procedures should be considered when deciding on the ideal intervention for UVFP. The purpose of this study is therefore to quantify the cost differences between MT and office-based IL in adults with UVFP.

## Methods

This economic analysis was conducted from the London Health Sciences Centre (LHSC) perspective-a tertiary academic hospital in Canada. All costs were reported in 2014 Canadian dollars. A 5-year time horizon was used. The discount rate was set at 5% to account for inflation and interest over time [8]. Time to relapse of IL was set to 1 year as the expected lifespan of calcium hydroxylapatite is between 1 to 2 years [4].

### Decision analytic model

**Fig. 1:**
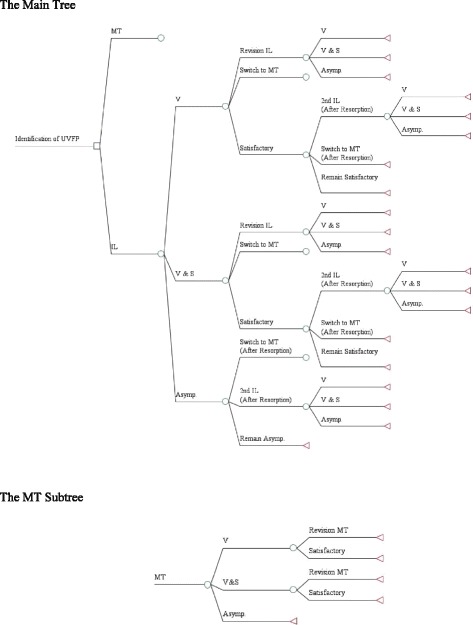
Decision Tree of Cost Minimization comparing Medialization Thyroplasty and Injection Laryngoplasty. The square represents the initial decision to undergo MT or IL after the identification of UVFP. Circles represent chance events, and triangles represent terminal nodes beyond which no further interventions and costs occurred. One month following the procedure (MT or IL), patients were stratified into 3 groups based on the post-procedural outcomes: voice symptoms (V), voice and swallowing symptoms (V & S), and asymptomatic (Asymp). There were no patients who complained of swallowing symptoms without voice symptoms. Patients with symptoms (V or V & S) after the implementation of initial IL have three possible paths: immediate revision IL (Revision IL), MT (Switch to MT), or observation if the patient was satisfied despite their symptomology (Satisfactory). Due to the temporary nature of the fillers used for IL, patients who were initially satisfied with their treatment despite symptomology could have three possible paths: repeat IL (2nd IL), MT (Switch to MT), or observation if the patient remained satisfied despite their symptoms (Remain Satisfactory). Similarly, patients who were asymptomatic after the initial IL could have three possible paths: relapse after the fillers are resorbed over time and have a repeat IL (2nd IL), undergo MT (Switch to MT), or remain asymptomatic (Remain Asymp). MT Subtree: For patients with symptoms (V or V & S) after MT, there were two possible paths: immediate revision MT (Revision MT), or observation if the patient was satisfied despite their symptomology (Satisfactory). For patients who were asymptomatic after MT, there was no further intervention as of MT is considered permanent

A decision tree model, consistent with the usual treatment pathways for MT and IL for patients presenting with UVFP, was developed to perform our cost-minimization analysis (Fig. 1). Analysis was conducted using TreeAge Pro 2009 software (TreeAge Software, Inc., Williamstown, MA).

### Medialization procedures

MT was performed in the operating room under light sedation and local anesthesia. A nasopharyngoscope was used for visualization of the vocal folds preoperatively and left in place intra-operatively to confirm medialization. The standard thyroplasty approach using the Montgomery® Thyroplasty Implant System (Boston Medical Products, Westborough, MA) was employed, as described by Montgomery and Montgomery [9]. Patients were brought to the post-operative recovery unit following the procedure. Patients were stratified by risk for post-operative complications as described by Zhao et al. and admitted or discharged home accordingly [10]. Reasons for admission include previous neck radiation or surgery, and other comorbidities such as myocardial infarction and stroke.

IL was performed in an outpatient, hospital-based Otolaryngology-Head and Neck Surgery clinic. The patient was positioned in the examination chair in the semi-recumbent position. The cricothyroid approach was most preferred, but transthyrohyoid membrane or transthyroid approaches were also used as indicated. Local anesthesia was infiltrated into the anterior neck soft tissues overlying the cricothyroid membrane and the airway. Under direct vision with the nasopharyngoscope, the needle was advanced to the thyroarytenoid muscle and an injection of calcium hydroxylapatite (Radiesse^TM^ Voice, Merz Aesthetics Inc., San Mateo CA) was completed until the paralyzed vocal fold was medialized. Patient vocalization confirmed medialization. The patient was monitored in the clinic waiting area for at least 30 min, then discharged home.

### Probabilities

Probabilities for model parameters were derived from a retrospective cohort of patients from LHSC. Patients were accrued after approval from the institution ethics review board (Institutional Research Ethics Board #105711). Consecutive patients treated by four Otolaryngology-Head and Neck surgeons with MT or IL for UVFP from April 2008 to April 2014 were eligible for inclusion. Other inclusion criteria were: 1) adult over the age of 18, 2) patients were medically eligible for both treatment modalities, and 3) at least one post-procedural follow-up. Patients were excluded if there was any possibility of resolution of UVFP as recovery may be mistaken for success of the procedure and skew probability of resolution of symptoms in the model. These included patients with idiopathic UVFP with a duration of less than 1 year at the time of presentation. Selection of MT versus IL was a joint decision by the surgeon and patient, considering patient preference and surgeon comfort. Date of decision to treat was set as the date of obtaining consent for the procedure.

Patient diagnosis, age, gender, and intervention were recorded. Post-procedural swallowing and voice symptoms were recorded at 1 month. Need for repeat or revision procedure and complications were assessed. Gastrostomy tube dependence rate was recorded where available.

#### Patient demographics

A total of 228 patients were screened for inclusion via retrospective review. One hundred four patients met the inclusion and exclusion criteria. Sixty-three patients underwent MT whereas 41 underwent IL. Patient demographics and clinical characteristics are reported in Table 1. Patient demographic characteristics were compared using the paired *t*-test and chi-squared test.

**Table 1:** Baseline Patient Demographics

	Medialization Thyroplasty	Injection Laryngoplasty	
Mean Age	61.5	70.4	*p* = 0.003^a^
Males (%)	37 (57.8%)	27 (63.9%)	*p* = 0.466
Voice Complaints (%)	63 (100%)	41 (100%)	*p* = 1.000
Swallowing Complaints (%)	28 (44.4%)	21 (51.2%)	*p* = 0.499
Etiology (N)	Idiopathic (15) Iatrogenic (23) Neoplastic (20) Traumatic (4) Stroke (1)	Idiopathic (3) Iatrogenic (13) Neoplastic (25)	

^a^statistically significant with α <0.05

#### Probabilities for medialization thyroplasty

Of the 63 patients initially treated with MT, 53 (84.1%) were asymptomatic at 1 month. Four patients continued to have voice complaints. Six patients reported both voice and swallowing symptoms. Two patients, one with voice complaints and one with voice and swallowing complaints, underwent revision MT. The patient with initial voice complaints was treated successfully with revision MT. However, the patient with initial voice and swallowing complaints remained symptomatic, underwent a second revision surgery, and was subsequently asymptomatic. As a second revision surgery is rare in the clinical setting, this was not included in the base case analysis, but the impact of its inclusion is discussed in the sensitivity analysis. Thirty-eight patients (60.3%) were admitted for overnight stay based on criteria of Zhao et al. [10] All patients undergoing revision MT were admitted due to prior neck surgery.

#### Probabilities for injection laryngoplasty

Forty-one patients underwent IL. Eighteen patients (43.9%) were asymptomatic at 1 month. Eight patients continued to have voice complaints and 15 patients continued to have voice and swallowing complaints at follow-up. Of these patients, three underwent repeat IL. Two of these patients were subsequently asymptomatic, but one continued to have voice complaints. This patient was consented for MT but passed away prior to the procedure due to a prior lung neoplasm.

Six patients had satisfactory initial results from IL but became symptomatic and underwent subsequent MT. Time from IL to MT was a mean of 18 months. All patients were asymptomatic following MT. No patients were admitted following IL.

### Costs

Where available, LHSC costs were utilized. Only direct medical care costs (costs for personnel, equipment, and materials) were considered in the base case analysis to reflect LHSC’s perspective. Indirect costs were included in the scenario analysis to provide a societal perspective.

#### Costs for medialization thyroplasty

Cost for MT consisted of four components: surgeon, anesthesiologist, operating room and recovery personnel, and equipment costs (Table 2). Surgeon and anesthesiologist costs were based on the schedule of benefits published by the Ontario Health Insurance Program (OHIP) [11]. Ontario operates as a single payer system and this government published list consists of uniform fees paid for physician services throughout the province. Operating room and recovery unit personnel costs were calculated by the duration of each procedure at LHSC’s rate of C$5.23/min. Material costs were based on the last 12 cases performed at the LHSC as prior data were not available. Total cost of MT was C$1440.11. Revision MT was assumed to cost the same as initial MT.

**Table 2:** Parameter Table - Base Case

Parameter	Base case	Reference
Probabilities ^a^ (%)		
Having a voice issue right after the initial MT	6.4	LHSC Data^d^
Undergoing revision MT for patients with voice issue after the initial MT	25.0	LHSC Data^d^
Having a voice and swallowing issue right after the initial MT	9.5	LHSC Data^d^
Undergoing revision MT for patients with voice and swallowing issue after the initial MT	16.7	LHSC Data^d^
Having a voice issue right after the initial IL	19.5	LHSC Data^4^
Having a voice and swallowing issue right after the initial IL	36.6	LHSC Data^d^
Undergoing revision IL for patients with voice issue after the initial IL	37.5	LHSC Data^d^
Having a voice issue after the revision IL for patients with voice issue after the initial IL	33.3	LHSC Data^d^
Switching to MT if asymptomatic right after the 1st IL but relapse over time	33.3	LHSC Data^d^
Admission after MT	60.3	LHSC Data^d^
Direct costs ($):		
Costs components for MT ^b^		
Surgeon	632.85	[11]
Nursing and OR aides	325.38	LHSC Data^d^
Supplies	316.42	LHSC Data^d^
Anesthesia	165.46	[11]
Inpatient stay if admitted after a MT ^c^	1,595.78	LHSC Data^4^
Costs components for IL		
Equipment CaHa)	305.71	LHSC Data^d^
Physician	256.11	[11]
Nursing staff	5.42	[12]
Discount rate	5%	[1]
Time to relapse	1 year	[2]

^a^ Only non-zero probabilities are listed. All probabilities for the remaining branches in the model are zero

^b^ Revision MT was assumed to cost the same as initial MT

^c^ Assumed rate of inpatient stay after MT is the same for patients with or without post-surgery symptoms

^d^ Based on London Health Sciences Centre (LHSC) retrospectively collected patient cohort

Inpatient stay following MT was C$1477.58/day based on the mean cost of postoperative stay at LHSC. Mean length of stay was 1.08 days. As 60.3% of all patients undergoing initial MT were admitted, an average inpatient stay cost of C$962.26 was included in the cost of MT. All revision MT patients were inpatients, and an average inpatient stay cost of C$1595.78 was added to the cost of revision MT.

#### Costs for injection laryngoplasty

The cost for IL consisted of three components: surgeon, personnel, and equipment costs (Table 2). Surgeon costs were determined by the OHIP Schedule of Benefits [11]. Personnel costs included clinic nursing costs. Average hourly wage as per the Ontario Nursing Association agreement was multiplied by the average length of time for injection (4.86 min) [12]. This was added to an average 5 min preparation time for room set up and clean up for a total duration of procedure of 9.86 min. All patients, regardless of undergoing MT or IL, would require initial consultation and consent in the clinic. Therefore, this cost was considered equal between both groups and not included in the cost calculations. Equipment costs were based on the institutional pharmacy and the healthcare materials management services department. Cost for the nasopharyngoscope and processing were obtained from the hospital and included processing time which was timed at an average of 6.5 min, multiplied by an average technician wage of C$22/h [13]. Following the procedure, all patients recovered in the clinic waiting area. This was during an active clinic and did not require any special personnel or equipment. Therefore, no cost was associated with patient recovery time. Total cost for an IL was C$567.24.

### Complications

One patient suffered from a postoperative hematoma following MT. This required a return visit to the operating room. While in the hospital, this patient experienced urinary retention requiring a longer hospital stay of 4 days. There were no complications following IL. Complication probabilities were set to zeros in the model in order to avoid undue influence of one special case in the MT arm.

### Sensitivity analysis

Deterministic and probabilistic sensitivity analyses were conducted to account for variability in the input parameters presented in Tables 3 and 4. One-way sensitivity analyses were conducted for all costs with a known range, for a discount rate of 3 and 10%, an IL effect duration of 2 years and the percent of patients undergoing repeat IL (Table 4).

**Table 3:** Probabilities for Sensitivity Analyses ^a^

Probabilities ^b^	Base case value (%)	Parameters for Beta distributions for PSA^c^	
		*α* ^d^	*β* ^e^
Having a voice issue right after the initial MT	6.4	4	59
Undergoing revision MT for patients with voice issue after the initial MT	25.0	1	3
Having a voice and swallowing issue right after the initial MT	9.5	6	57
Undergoing revision MT for patients with voice and swallowing issue after the initial MT	16.7	1	5
Having a voice issue right after the initial IL	19.5	8	33
Having a voice and swallowing issue right after the initial IL	36.6	15	26
Undergoing revision IL for patients with voice issue after the initial IL	37.5	3	5
Having a voice issue after the revision IL for patients with voice issue after the initial IL	33.3	1	2
Switching to MT if asymptomatic right after the 1st IL but relapse over time	33.3	6	12
Admission after MT	60.3	38	25
Having a voice issue right after the initial MT	4.3 [15]		

^a^ All probabilities are derived from the LHSC data set except the probability of having voice issue right after the initial MT

^b^ Only non-zero probabilities are listed. All probabilities for the remaining branches in the model are zero

^c^ PSA: probabilistic sensitivity analysis

^d^ The parameter *α* for beta distribution equals the number of occurrence

^e^ The parameter *β* for beta distribution equals the difference of sample size and number of occurrence

**Table 4:** Parameters used for Sensitivity and Scenario Analysis

Other Parameters	Base Case	Low Value	High Value	Reference
Discount rate	5%	3%	10%	[1]
Time to relapse	1 year	1 year	2 years	[2]
Proportions of different age groups ^a^:				
Proportion of age 15–24	1.0%			LHSC Data^b^
Proportion of age 25–54	22.1%			LHSC Data^b^
Proportion of age 55–65	24.0%			LHSC Data^b^
Proportion of age 66+	52.9%			LHSC Data^b^

^a^ The proportions of different age groups are used in the scenario analysis only in order to calculate the productivity loss as the indirect costs

^b^ Based on London Health Sciences Centre (LHSC) retrospectively collected patient cohort

Scenario analysis was conducted to investigate the societal cost of MT versus IL. Loss of productivity while awaiting a procedure for UVFP was calculated as an indirect cost. Median wait time was used for scenario analysis as the range of wait times was wide (9-330 days). The average wage for each age group was calculated according to Statistics Canada assuming an average 8 h/day [14]. Assuming no patients could work during wait time for their procedure, the estimated productivity loss is reported in Table 5. Roy et al. found that 4.3% of patients were unable to work with their voice disturbance [15]. Productivity losses were calculated based on age-weighted average according to the proportions in Table 4. Productivity loss for MT was C$507.96 and IL C$26.58. A second scenario analysis was conducted to account for the impact of the patient who underwent two revision MTs.

**Table 5:** Costs in 2014 Canadian Dollars for Sensitivity Analyses

Costs	Base Case	Standard Deviation	One-Way Sensitivity Analysis		Parameters for Gamma distributions for PSA		References
			Low Value	High Value	*α* ^a^	*β* ^b^	
Direct costs:							
Costs components for MT ^c^							
Surgeon	632.85						[11]
Nursing and OR aides	325.38	104.30	118.49	597.61	9.7315	0.0299	LHSC Data^f^
Supplies	316.42	175.44	167.70	525.11	3.2529	0.0103	LHSC Data^f^
Anesthesia	165.46	32.72	118.28	266.14	25.5637	0.1545	[11]
Inpatient stay if admitted after MT ^d^	1,595.78	635.36			6.3083	0.0040	LHSC Data^f^
Costs components for IL							
Equipment (CaHa)	305.71		285.97	325.45			LHSC Data^f^
Physician	256.11						[11]
Nursing staff	5.42	10% of the mean mean			100	18.4577	[12]
Indirect costs ^e^:							
Productivity loss for MT if all patients cannot work during waiting time							
Age 15–24	29666.40						[14]
Age 25–54	32913.36						[14]
Age 55–65	17677.44						[14]
Age 66+	0						[14]
Productivity loss for IL if all patients cannot work during the waiting time							
Age 15–24	0						[14]
Age 25–54	2796.56						[14]
Age 55–65	0						[14]
Age 66+	0						[14]

^a^ The parameter ***α*** for a gamma distribution is calculated by *mean* ^2^/*variance*

^b^ The parameter *β* for a gamma distribution is calculated by *variance*/*mean*

^c^ Revision MT was assumed to cost the same as initial MT

^d^ We assumed inpatient stay if admitted after a MT is the same for patients with or without post-surgery symptoms

^e^ The base case values of indirect costs are used in the scenario analysis only

^f^ Based on London Health Sciences Centre (LHSC) retrospectively collected patient cohort

Probabilistic sensitivity analysis using a Monte Carlo simulation was performed on direct costs. A beta distribution was used for each probability (Table 3) while a gamma distribution was used for each cost component (Table 5).

## Results

### Base case analysis

Probabilities and costs associated with the base model are presented in Table 2. Base case analysis showed that patients treated with MT was C$2499.10, compared to C$943.19 when treated with IL. Cost savings with the IL procedure was C$1555.91 per patient compared to MT.

### Sensitivity analyses

#### One-way sensitivity analysis

**Fig. 2:**
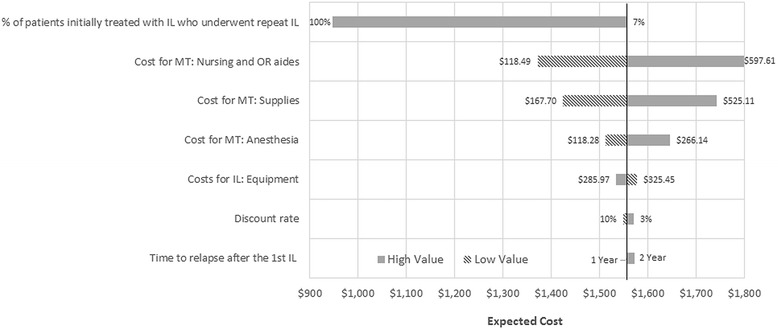
Tornado Diagram Demonstrating Results of the One-Way Sensitivity Analysis. Abbreviations: IL:injection laryngoplasty; MT: medialization thyroplasty; OR: operating room

Costs with variances are summarised in Table 5. Parameters compared for sensitivity analysis including discount rate and relapse rate are summarised in Table 4. Cost savings ranged from C$974.00 to C$1799.00, all favouring IL. These savings persisted even if all patients initially undergoing IL underwent a repeat IL. All one-way sensitivity analyses results are presented in Fig. 2.

#### Scenario analysis

Scenario analysis was conducted to account for the impact of productivity loss during the wait time for treatment. Assuming a 4.3% inability to work, cost of MT was C$3007.07 compared to C$1042.43 for IL. Cost savings was C$1964.64. Second scenario analysis accounting for the patient who underwent two revision MTs found the cost for initial treatment with MT increased to C$2547.27 and the cost savings increased to C$1604.08.

#### Probabilistic sensitivity analysis

**Fig. 3:**
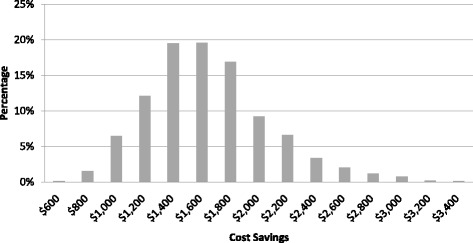
Results of Probabilistic Sensitivity Analysis: Distribution of Cost Savings. Generated from cost savings (when switching from initial treatment with medialization thyroplasty to initial treatment with injection laryngoplasty) from 1000 trials in the probabilistic sensitivity analysis

Parameters utilized to derive beta distributions and gamma distributions are presented in Tables 3 and 5, respectively. Minimum cost savings with the IL procedure was C$596 while the maximum cost savings was C$3626. The distribution of cost savings of the probabilistic sensitivity analyses is presented in Fig. 3.

## Discussion

Many different treatment options exist in the Otolaryngologist-Head and Neck surgeon’s armamentarium for UVFP. MT and IL both have the common goal of medialization of the affected vocal fold to facilitate contact with the unaffected side. Though several studies investigated the efficacy of both techniques, this is, to our knowledge, the first direct comparison of costs between MT and IL procedures. This study shows that there are cost savings when patients are treated initially with IL with a potential cost savings of C$1555.91 per patient. Sensitivity analyses demonstrated the robustness of this conclusion, with probabilistic sensitivity analysis showing cost savings from C$596 to C$3626 all favouring the IL procedure. Hillel et al. published a study investigating costs differences in patients undergoing laser photoablation of laryngeal lesions in the endoscopy suite versus the operating room and found a result of significant cost savings in the endoscopy room setting [16]. This bolsters our finding that moving procedures from the operating room to the outpatient setting results in significant cost savings.

In this cohort of patients, only 43.9% of patients were asymptomatic after IL. This cohort includes the cohort of patients first treated at LHSC with IL. Laryngeal injection may not be a familiar technique to some surgeons and the lower success rate may be due to a learning curve for IL. As well, persisting voice complaints despite adequate IL may be due to other aspects of voice production such as breath support as neoplastic causes for UVFP (such as lung cancer) represented the majority of included patients. Rosen et al. reported on their findings in a multi-centred prospective trial of IL with calcium hydroxylapatite in patients with glottic insufficiency [17]. Of the 63 patients treated, 81% patients reported their voice to be significantly, greatly, or somewhat improved at 12 months. However, only 36 of those patients had a diagnosis of UVFP. Improvements in the success of IL would likely decrease the need for revision procedures, further decreasing the cost of initial treatment with IL. Due to the possible variability of operator success with IL, sensitivity analysis was completed demonstrating that even if 100% of patients treated with IL required a repeat injection, initial treatment with IL would continue to be more economical.

The lifespan of calcium hydroxylapatite is between 1 to 2 years. In this study, mean time from IL to further treatment was 18 months, which is consistent with the findings by Shen et al. [4] Our base case analysis and sensitivity analysis analyzed costs for both a 1 and 2 year lifespan, demonstrating cost savings with patients undergoing IL in both scenarios. In this cohort, 66.7% patients who were initially asymptomatic after IL did not require further treatment within a 5-year time horizon. Prendes et al. found that those with UVFP had effects of IL with a temporary agent longer than the expected lifespan of the injected material [18]. The mechanism of this lasting effect is unclear. However, considering many patients present with UVFP due to neoplastic processes or injury during treatment of neoplastic processes, the 2 year or longer treatment effect may be an adequate length of effectiveness for many patients. It is also possible that the gradual resorption of the injected material allows for compensation from the contralateral vocal fold in some patients, alleviating the need for repeat treatments.

### Complications

Only one complication occurred in our cohort. Due to the unusual nature of this complication, it was not included in our analysis. As the only complication occurred in the MT group, inclusion of the costs of this complication would not have changed the main conclusions. Abraham et al. found a 14% complication rate in MT, including transient edema, hematoma, infection, extrusion, and airway issues [19]. Mathison et al. found that 19.1% of patients had complications from IL, including aborted procedure, vasovagal reaction, rapid resorption of the injected material, vocal fold hemorrhage, and superficial lamina propria injection. All these complications were self-limited and did not require hospitalization, limiting their associated costs [20].

### Limitations and future directions

This study relies on patient-reported symptoms of voice or swallowing complaints. Patients were treated by four surgeons and data were accrued retrospectively, therefore objective measures such as maximum phonation time or Voice Handicap Index were not reliably recorded. However, in this cost analysis, the major determinant of increased cost is the need for a second procedure-the necessity of which is primarily driven by the subjective complaints of the patient. Therefore, while imperfect, patient-reported outcomes are a useful predictor for cost in a study such as this.

Probability parameters rely on the institutional cohort. This allows for increased homogeneity of the data but it limits the sample size. A literature search of studies reporting outcomes following MT or IL showed significant variability in the reported outcome measures and not all parameters were available. As a result of the smaller sample size, many of the probabilities on the decision tree were set to zero.

A larger cohort of patients would allow for capturing rare complications and the associated transition probabilities as well as costs. Our cohort also included many aetiologies for UVFP. Therefore, these findings should be further validated in other UVFP presentations and patient populations. As well, differing techniques in IL and MT or other management strategies may yield different results. In particular, the patients in this study did not have access to voice therapy with a speech language pathologist (SLP) through LHSC and thus would need to seek SLP services outside of our institution. Therefore, costs related to SLP services were not included in our analysis. Applicability of our findings to a practice setting with access to SLP services may be limited.

A longer follow-up period and time horizon would potentially capture more treatment failures and revision procedures to determine the long-term costs of MT versus IL. However, many of the patients included in our cohort developed UVFP due to neoplastic processes, limiting the duration of follow-up.

This study focused on institutional costs of IL and MT procedures. Indirect or societal costs were not available, hence we investigated the influence of productivity losses using aggregate data from Statistics Canada. Although our study is unable to offer definite conclusions regarding societal cost-savings, it is still relevant from a hospital perspective as the direct costs are relevant to hospital’s budget. All costs and parameters in this model were obtained from the LHSC perspective, part of a single payer Canadian system. While this increases the uniformity of costs between patients and the results are likely to be similar across Canadian hospitals, replicability of these findings in different payer systems may be limited. As well, all surgeons at this centre utilized the Montgomery® Thyroplasty Implant System for MT and calcium hydroxylapatite for IL. Different equipment preferences at different centres may yield varying results. However, our findings showed large cost savings in favour of IL, the robustness of which was shown in our probabilistic sensitivity analysis.

## Conclusions

Based on this cost-minimization analysis, initial treatment with IL results in C$1555.91 more cost savings per patient than initial treatment with MT at a 5-year time horizon in the base case analysis. Deterministic and probabilistic sensitivity analyses and a scenario analysis are consistent in showing that initial treatment with IL is less costly than MT. Specifically, switching from MT to IL will result in a minimum cost savings of C$596 per patient. Though our findings reflect the experience at a single Canadian centre, we expect these cost savings to be corroborated in a variety of different environments given the marked difference in cost between MT and IL.

## Abbreviations

IL: Injection laryngoplasty
LHSC: London Health Sciences Centre
MT: Medialization thyroplasty
OHIP: Ontario Health Insurance Plan
UVFP: Unilateral vocal fold paralysis
